# Associations of Childcare Arrangements with Adiposity Measures in a Multi-Ethnic Asian Cohort: The GUSTO Study

**DOI:** 10.3390/ijerph182212178

**Published:** 2021-11-19

**Authors:** Beverly Wen-Xin Wong, Jia Ying Toh, Ray Sugianto, Airu Chia, Mya Thway Tint, Wen Lun Yuan, Natarajan Padmapriya, Carla Lança, Seang-Mei Saw, Yung Seng Lee, Lynette Pei-Chi Shek, Kok Hian Tan, Fabian Yap, Keith M. Godfrey, Yap-Seng Chong, Falk Müller-Riemenschneider, Johan G. Eriksson, Shiao-Yng Chan, Mary Foong-Fong Chong

**Affiliations:** 1 Singapore Institute for Clinical Sciences, Agency for Science, Technology and Research (A*STAR), Singapore 117609, Singapore; obgwxbw@nus.edu.sg (B.W.-X.W.); toh_jia_ying@sics.a-star.edu.sg (J.Y.T.); mya_thway_tint@sics.a-star.edu.sg (M.T.T.); wenlun.yuan@inserm.fr (W.L.Y.); paeleeys@nus.edu.sg (Y.S.L.); obgcys@nus.edu.sg (Y.-S.C.); obgjge@nus.edu.sg (J.G.E.); obgchan@nus.edu.sg (S.-Y.C.); 2 Saw Swee Hock School of Public Health, National University of Singapore, 12 Science Drive 2, Singapore 117549, Singapore; ray.sugianto@u.nus.edu (R.S.); airu-chia@nus.edu.sg (A.C.); obgnp@nus.edu.sg (N.P.); ephssm@nus.edu.sg (S.-M.S.); falk.m-r@nus.edu.sg (F.M.-R.); 3 Department of Obstetrics & Gynaecology, Yong Loo Lin School of Medicine, National University of Singapore, 1E Kent Ridge Road, Singapore 119228, Singapore; 4 Inserm, INRAE, CRESS, Université de Paris, F-75004 Paris, France; 5 Singapore Eye Research Institute, 20 College Rd, Singapore 169856, Singapore; carla.costa@estesl.ipl.pt; 6 Escola Superior de Tecnologia da Saúde de Lisboa (ESTeSL), Instituto Politécnico de Lisboa, 1549-020 Lisbon, Portugal; 7 Comprehensive Health Research Center (CHRC), Escola Nacional de Saúde Pública, Universidade Nova de Lisboa, 1099-085 Lisbon, Portugal; 8 Graduate Medical School, Duke-National University of Singapore, Singapore 169857, Singapore; tan.kok.hian@singhealth.com.sg (K.H.T.); fabian.yap.k.p@singhealth.com.sg (F.Y.); 9 Department of Pediatrics, Yong Loo Lin School of Medicine, National University of Singapore, 1E Kent Ridge Road, Singapore 119228, Singapore; lynette_shek@nuhs.edu.sg; 10 Division of Paediatric Endocrinology, Khoo Teck Puat-National University Children’s Medical Institute, National University Hospital, National University Health System, 5 Lower Kent Ridge Rd, Singapore 119074, Singapore; 11 Department of Pediatric Allergy, Immunology, and Rheumatology, Khoo Teck Puat-National University Children’s Medical Institute, National University Hospital, National University Health System, 5 Lower Kent Ridge Rd, Singapore 119074, Singapore; 12 Department of Maternal Fetal Medicine, KK Women’s and Children’s Hospital, 100 Bukit Timah Rd, Singapore 229899, Singapore; 13 Department of Pediatric Endocrinology, KK Women’s and Children’s Hospital, 100 Bukit Timah Rd, Singapore 229899, Singapore; 14 Lee Kong Chian School of Medicine, Nanyang Technological University, 59 Nanyang Drive, Singapore 636921, Singapore; 15 Medical Research Council Lifecourse Epidemiology Unit, University of Southampton, Tremona Rd, Southampton SO16 6YD, UK; kmg@mrc.soton.ac.uk; 16 Southampton Biomedical Research Centre, National Institute for Health Research, University Hospital Southampton, NHS Foundation Trust, Tremona Rd, Southampton SO16 6YD, UK; 17 Folkhälsan Research Center, Topeliuksenkatu 20, 00250 Helsinki, Finland; 18 Department of General Practice and Primary Health Care, Helsinki University Hospital, University of Helsinki, Tukholmankatu 8 B, 00290 Helsinki, Finland

**Keywords:** childcare, adiposity, preschool, caregiver type, Asian

## Abstract

Childcare arrangements shape behavioural patterns that influence the risk of childhood obesity. However, little is known of its influence on childhood obesity in Singapore. We aim to examine the associations between childcare arrangements at the age of 5 years and childhood adiposity at age 6 years. Children from the GUSTO study were grouped into three childcare arrangements at age 5: full-time centre-based childcare (FC), partial centre-based with parental care (PCP), and partial centre-based with non-parents (grandparents and domestic helpers) as caregivers (PCN). Diet, physical activity and sedentary behaviour information were collected at age 5, while anthropometric measurements were collected at age 6. Associations were analysed using multivariable regression models. Among 540 children, those in PCN had higher BMI *z*-scores (*β*: 0.34; 95% CI: 0.01, 0.66), greater sum of skinfold thicknesses (mm) (*β*: 3.75; 95% CI: 0.53, 6.97) and were 3.55 times (95% CI: 1.78, 7.05) more likely to be overweight/obese than those in FC. Adiposity measures in PCP children did not differ from those in FC. PCN children were reported to have more screen time and greater fast-food intake. Children in PCN tended to have higher adiposity measures. Greater engagement of non-parental caregivers should be considered in interventions targeting child obesity.

## 1. Introduction

Globally, overweight and obesity rates have surged among preschool children, from 4.2% to 6.7% over a span of 20 years [1]. In 2019, 3% of Southeast Asian children below 5 years of age were found to be overweight [2]. The prevalence of overweight and obesity in Singaporean preschool children was 7.0% and 5.3%, respectively, in 2013 [3]. Children with obesity are more likely to become adults with obesity and have an increased risk of developing several non-communicable diseases such as diabetes and cardiovascular disease in later life [4,5,6].

Obesity can be attributed to an imbalance in energy intake and expenditure [7], with physical activity, dietary intake and sedentary behaviour being major risk factors [8]. The early years are critical in the development and establishment of eating habits [9] and physical activity levels [10]. These behavioural patterns are crucially shaped by the environment [11], such as the childcare setting and type of caregiver, especially since 80% of preschool-aged children in developed nations are in some form of non-parental childcare [12]. In Singapore, rising childcare demand can be seen from the exponential increase in the number of childcare centres from 98 centres in 1986 [13] to over 1500 centres in 2020 [14].

Common childcare arrangements referred to in the literature include (i) parental care, (ii) informal care, referring to care mainly provided for by relatives (e.g., grandparent) or non-relatives (e.g., neighbours and friends) [15] and (iii) centre-based care, referring to regulated care settings [16] such as day care and preschools [17]. Recent systematic reviews on childcare arrangements concluded that informal care was associated with a higher likelihood of childhood overweight/obesity than parental care [15,16]. Geoffroy et al. demonstrated in the Quebec Longitudinal Study of Child Development that children between 1.5 to 4 years of age cared for by a relative had higher odds of being overweight at 4 to 10 years of age than parental care [18]. Similarly, a longitudinal study conducted in Japan observed that boys who were cared for by grandparents at 3 years of age were more likely to be overweight at 6 and 12 years of age [19]. Further meta-analysis found that grandparental childcare increased the risk of childhood obesity by 30% [20]. These studies suggested the increased risk of childhood obesity could be due to informal caregivers’ poor knowledge in healthy eating, preference for indulgent feeding and influence on children’s physical activity [15,16,18,19,20].

When comparing children attending centre-based childcare with those who did not, findings on the risks of overweight/obesity were mixed. A few studies reported increased risks when attending centre-based childcare [18,21,22,23,24], several others reported decreased risks [25,26,27,28,29] while some found no association [30,31,32,33,34]. Suggested explanations for some of these findings were differences in nutrition and physical activity regulation and quality in preschools [18]. Conversely, other studies cited factors such as enhanced nutrition and higher rates of health screening [27] in centre-based childcare as reasons in ameliorating obesity risks.

Given the inconsistent findings as described above, we aimed to examine the associations between childcare arrangements and adiposity measures in a multi-ethnic Asian cohort. We also aimed to explore differences in lifestyle behaviours such as dietary intake, physical activity and sedentary behaviour between children in different childcare arrangements. Given that local childcare centres are required to comply with certain dietary guidelines under the Healthy Meals in Child Care Centres Programme (HMCCP) [35], we hypothesised that children in full-time centre-based childcare would have lower prevalence of adiposity compared to children in partial centre-based care. Findings from this study would broaden our understanding of childcare arrangements in Singapore and their associations with childhood obesity and lifestyle behaviours. This could elucidate potential correlates that can be targeted for childhood obesity interventions.

## 2. Materials and Methods

### 2.1. Study Background and Design

Participants were part of the Growing Up in Singapore Towards healthy Outcomes (GUSTO) study, an ongoing longitudinal mother–offspring cohort study examining influences in early development on metabolic and growth outcomes in women and children. Pregnant women in their first trimester of pregnancy, of Chinese, Malay or Indian ethnicities and attended their first antenatal ultrasound scans at either the KK Women’s and Children’s Hospital (KKH) or National University Hospital (NUH) were screened between June 2009 to September 2010. Mothers were of a homogenous ethnicity with the intention of residing in Singapore for the next 5 years and did not have serious pre-pregnancy health conditions such as type I diabetes. Both mother and child were followed up from birth and the inclusion and exclusion criteria can be found in greater detail in Soh et al. [36]. Data from children at 5 and 6 years of age were used for the current study. This study was approved by the Institutional Review Board of KKH and NUH and was registered with ClinicalTrials.gov as NCT01174875. Written informed consent was obtained from all mothers in accordance with the Declaration of Helsinki [37].

### 2.2. Assessment of Childcare Arrangement at Age 5

At the age of 5 years, children’s caregivers were asked to complete an interview-administered questionnaire during the clinic visit. This questionnaire consisted of several questions regarding childcare arrangements. They included: (1) attendance at childcare centres (yes/no), (2) number of days spent per week in childcare centres (0.5 day to 7 days), (3) duration spent per day in childcare centres (full-day/half-day/less than half-day) and (4) age of childcare centre commencement (in months). The questionnaire also included questions related to the primary caregiver (mother, father, grandparent/relative, domestic helper, or shared responsibility between more than one caregiver).

Childcare arrangements were grouped based on the amount of time spent in centre-based childcare. Children who attended centre-based childcare for at least five full days a week were grouped under full-time centre-based childcare (FC). Children who attended centre-based childcare for four or less full days a week or any number of half days/less than half days a week were grouped under partial centre-based childcare (PC). The PC group was further sub-categorised based on the main person caring for the child at home (primary caregiver type)—PC parent (PCP) or PC non-parent (PCN) (e.g., grandparent/relative or domestic helper).

### 2.3. Dietary Measures at Age 5

Dietary intakes of children were ascertained using a validated food frequency questionnaire (FFQ) [38], administered by an interviewer to caregivers during the 5-year clinic visit. Caregivers reported the child’s frequency of consumption and portion sizes of 112 food items, along with frequency of consuming meals purchased outside of home, in the previous month. Further details can be found elsewhere [38]. Similar food items in the FFQ were combined to form seven key food groups of interest [39]: (1) deep-fried foods, (2) fast foods, (3) fruits, (4) sugar-sweetened beverages (SSB), (5) sweet snacks, (6) vegetables and (7) whole grains (Table S1).

Frequency of snacking was also reported in a separated feeding practices questionnaire (“snacks all day and has no real meals”, “snacks all day but has real meals”, “snacks occasionally during tea time and does not snack much”).

### 2.4. Physical Activity, Sedentary Behaviour and Screen Time Measures at Age 5.5

Movement behaviour measures such as physical activity, sedentary behaviour and screen time were measured using a validated preschool age physical activity questionnaire (pre-PAQ) [40] during the 5.5-year clinic visit. Children’s caregivers reported the total amount of time spent per day by the child on physical activity and moderate-to-vigorous physical activity (MVPA). Sedentary behaviour (e.g., napping, sitting while eating meals, watching television (TV)/videos, playing games or reading books and sitting in motorised transport while travelling) and screen time (e.g., watching TV, using mobile devices, computers and video game consoles) were also reported by their caregivers. Details have been previously described [41].

### 2.5. Adiposity Measures at Age 6

During their clinic visit at age 6 years, children had their weight, height and skinfold thicknesses (triceps, biceps, subscapular and supra-iliac) measured using a standardised protocol, as detailed previously [42]. Weight and standing height, with the removal of shoes and inclusion of light clothing, were measured in duplicate using a weighing scale (SECA 803) and stadiometer (SECA stadiometer 213), respectively. The average of these measurements was then calculated. BMI (kg/m^2^) was converted into *z*-scores specific for age and sex in accordance to the World Health Organisation’s Child Growth Standards [43]. The cut-off for children who were overweight/obese was +1 SD above the reference distribution [43]. Skinfold thicknesses were measured in triplicates from the right side of the body using Holtain skinfold calipers (Holtain, Ltd., Crymych, UK), and the average of these measurements was calculated. Sum of skinfold thicknesses was calculated by taking the sum of the four averaged skinfold measurements.

### 2.6. Covariates

Age, ethnicity, education attainment, household income and occupation of mothers were collected at recruitment. During the 6-year clinic visit, maternal total physical activity and BMI (kg/m^2^) was obtained via self-reported physical activity questionnaire and calculated from height (SECA stadiometer 213) and weight (SECA 803) measurements respectively. Child sex and birth order were obtained from delivery records, while birthweight and total skinfold were measured at birth.

### 2.7. Statistical Analysis

Differences in demographic variables among childcare arrangements were analysed using Pearson’s chi-square test and one-way analysis of variance (ANOVA) with Bonferroni post-hoc test. Frequencies of missing demographic data were between <1% to 10%. Missing values for total skinfold at birth (*n* = 21); maternal education attainment (*n* = 3); maternal employment (*n* = 12); household income (*n* = 34); maternal physical activity 6 years postpartum (*n* = 23); maternal TV viewing 6 years postpartum (*n* = 24) and maternal BMI 6 years postpartum (*n* = 47) were imputed 20 times using multiple imputation by chained equations [44], and the results were pooled.

Differences in dietary food groups and movement behaviour measures among childcare arrangements were presented as median (IQR) and analysed using the Kruskal–Wallis test with a two-sided test of equality for column proportions. Whereas differences in snacking and eat out frequencies among childcare arrangements were presented as *n* (%) and analysed using Pearson’s chi-square test.

Multivariable general linear model (GLM) was used to analyse differences in adiposity measures between childcare arrangements, PCP and PCN, with the reference group, FC. Potential confounders were identified a-priori from previous studies [15,16,31]. When examining child BMI *z*-scores and sum of skinfold thicknesses, the models were adjusted for age of childcare commencement, birth order, ethnicity, maternal age, maternal education, maternal employment, maternal BMI 6 years postpartum and maternal physical activity level 6 years postpartum. Binary logistic regression model was used to estimate the likelihood of being overweight/obese among PCP and PCN children compared to FC children, with the same confounders adjusted for as the GLM.

All analyses were performed using SPSS version 26.0 (IBM Inc., Chicago, IL, USA).

## 3. Results

### 3.1. Study Sample Characteristics

**Figure 1:**
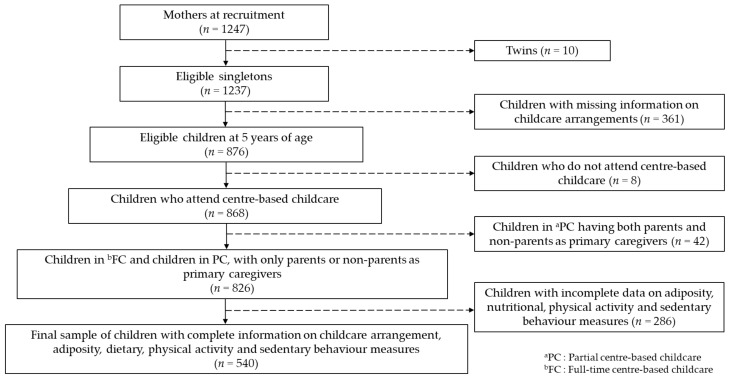
Participant flow diagram (*n* = 540).

Out of 1247 children recruited, we excluded twins (*n* = 10) and children with incomplete information on childcare arrangements (e.g., caregivers did not answer questions on duration spent per day in childcare) at 5 years of age (*n* = 361). A small number of children was reported not attending any centre-based childcare (*n* = 8) and was excluded from our analysis due to lack of statistical power to analyse them as a group. Children attending partial centre-based childcare with both parents and non-parents as primary caregivers were further excluded (*n* = 42) to ensure homogeneity in primary caregiver responsibility. Our final sample comprised of 540 children who had complete information on adiposity, dietary, physical activity and sedentary behaviour measures (Figure 1), of which 50.4% were under FC, 35.5% under PCP and 14.1% under PCN.

Out of those in PCP, mothers were the most common primary caregiver (96.4%), followed by fathers (2.6%) and both parents (1.0%). For those in PCN, grandparents (51.3%) and domestic helpers (43.4%) represented the majority of primary caregivers, while other relatives such as aunty (2.6%) and shared responsibilities between grandparents and domestic helpers (2.6%) made up the rest (Table S2).

Among the 540 children included, there was an equal mix of both sexes. More children in PCP were the second child and above compared to children in FC and PCN. More children in FC commenced childcare at a relatively earlier age (<24 months) compared to those in PCP and PCN (Table 1).

Fewer Chinese mothers had children in PCP compared to FC and PCN, while more Malay and Indian mothers had children in PCP compared to FC. PCP mothers tended to be older than PCN mothers. They also had lower educational attainment, lower household income and were mostly unemployed. At 6 years postpartum, PCP mothers tended to be overweight/obese compared to FC and PCN mothers, whereas more PCP mothers reported spending 150 min or more per week on physical activity than FC mothers (Table 1).

Children excluded from the analysis, due to missing information on childcare arrangement at age 5, physical activity, sedentary behaviour, screen time and adiposity or did not attend centre-based childcare or had non-homogeneous primary caregiver responsibility (*n* = 697), had a lower birth weight (mean ± SD: 3.0 ± 0.5 kg vs. 3.1 ± 0.4 kg) and fewer of them started childcare at a young age (commenced before 25 months: 28.4% vs. 33.8%) than those included. In addition, excluded mothers were younger as compared to those included (mean ± SD: 30.0 ± 0.5 years vs. 31.1 ± 5.2 years) (Table S3). Similar findings were observed when comparing participants with and without childcare arrangement data (*n* = 876 and *n* = 361 respectively) (Table S4).

### 3.2. Dietary Measures

The consumption of whole grains was higher among PCP children (median: 11.2 g/day) compared to FC children (median: 0.0 g/day). Deep fried food consumption was higher among PCP (median: 25.7 g/day) and PCN (median: 22.1 g/day) children compared to FC (median: 17.5 g/day) children. Additionally, fast food consumption was higher among PCN children (median: 17.9 g/day) compared to FC children (median: 12.9 g/day) (Table 2).

More children in PCP (24.0%) and PCN (22.7%) snacked throughout the day compared to children in FC (13.0%). No other significant differences were found for fruits, vegetables, sugar-sweetened beverages and sweet snacks consumption, as well as eating out frequency among children of different childcare arrangements (*p* > 0.05) (Table 2).

### 3.3. Movement Behaviour Measures

PCN children spent more time engaging in sedentary behaviour per day (median: 260.4 min/day) compared to FC children (median: 187.9 min/day). PCN children also had higher amounts of daily screen time (median: 115.7 min/day) compared to FC (median: 68.6 min/day) and PCP (median: 77.1 min/day) children (Table 3).

No significant differences in physical activity measures were found among children of different childcare arrangements (*p* > 0.05) (Table 3).

### 3.4. Adiposity Measures

In adjusted models, PCN children were associated with 0.34 SD higher BMI *z*-score (95% CI: 0.01, 0.66) and 3.75 mm greater sum of skinfold thicknesses (95% CI: 0.53, 6.97) at age 6 years compared to FC children (Table 4). In addition, PCN children were 3.55 times (95% CI: 1.78, 7.05) more likely to be overweight/obese compared to FC children (Table 5).

No other significant associations were found in PCP children when compared to FC children (*p* > 0.05) (Table 4 and Table 5).

## 4. Discussion

In line with our hypothesis, we found that preschool children in partial centre-based childcare with non-parents as primary caregivers were associated with higher adiposity and more likely to be overweight/obese compared to preschool children in full-time centre-based childcare. This suggests that a longer duration spent in centre-based childcare could have an inverse association with adiposity status in preschool children. Our findings on poorer diet quality and longer time spent in sedentary behaviour among preschool children in partial centre-based childcare provided further evidence to support this association.

The protective effects of centre-based childcare have been demonstrated previously [25,26,27,28,29]. Belfield et al. reported that children attending centre-based childcare were at a lower risk of childhood obesity due to lower probabilities of soda and chips consumption and a higher probability of fruits consumption compared to those not in centre-based care [27]. In another study, Robson et al. reported that children consumed less fruit and vegetables outside of centre-based childcare and overweight/obesity status was positively associated with energy intake away from centre-based childcare [45]. Contrary to these findings, other studies reported associations of centre-based childcare with overweight/obesity risks [18,21,22,23,24]. McGrady et al. explained that very few preschool settings in the United States met dietary guidelines, resulting in the consumption of more calories than needed [24]. A Canadian birth cohort study attributed the increased overweight/obesity risks to the broad and vague nature of physical activity and nutrition guidelines, making it a challenge for childcare settings to implement them practically [18]. This was also supported by Gubbels et al. where they suggested differences in childcare systems and quality of childcare could contribute to the conflicting findings [23]. Hence, it seems that the extent of childcare centre regulation by local authorities is crucial in curbing unnecessary weight gain among preschool children.

In Singapore, the Healthy Eating in Child Care Centres Programme (HECCP) was implemented in 2010 and childcare centres have the option to participate in it [13,46]. Participating childcare centres had to limit servings of deep-fried foods to once per week and provide 0.5–1 serving of fruits and vegetables each per day [46]. Implementation of these guidelines plausibly accounted for lower fast food and lower deep fried food intakes among FC children. However, whole grain consumption among FC children were the lowest at median intake of 0 g/day. Adequate whole grain consumption was not part of the 2010 guidelines but was revised in the 2016 HMCCP to include the use of whole grains in sandwiches and rice/porridge [35]. Dietary data in this study were collected in 2015, before the revised guidelines were implemented, hence possibly accounting for the low whole grain intake. The non-participation of some childcare centres in HECCP could also have accounted for low whole grain consumption, hence stressing the need for more childcare centres to be involved in such government initiatives.

Conversely, the promotion of whole grains consumption to the general population through multiple campaigns was introduced by the Singapore’s Health Promotion Board in 2009 and in the years that followed. This likely resulted in an increased consumption of unrefined carbohydrates (as a proportion of total carbohydrates) in Singaporean adults from 14% to 17% between 2010 to 2018 [47]. Previous findings from the GUSTO study also observed that mothers who modelled healthy food intake as part of their feeding practice had children with higher whole grain consumption at 5 years of age [39]. Hence, these factors could account for the higher consumption of whole grains in children partially care for by parents (PCP).

Our findings that non-parental care was associated with higher adiposity concur with earlier studies [19,20]. A Japanese study reported high frequencies of eating between meals when cared for by grandparents at 3 years of age and a higher BMI at 6 years of age [19]. Similarly, we found that more PCN and PCP children snack throughout the day. Snacking has been associated with an increase in energy intake, with sweets and salty snacks as common food items [48]. Furthermore, Li et al. reported increased consumption of sugar-added drinks and unhealthy snacks among children cared by grandparents [49]. Qualitative findings from the same study suggested that grandparents tend to have misconceptions that fat children are healthy and high energy or fat content foods are nutritious [49], hence possibly encouraging the consumption of more snacks and oily foods among their grandchildren. The findings from Li et al. could plausibly explain the high fast food and deep-fried food intakes among PCN children. In addition, a lack of awareness of health recommendations among grandparents and domestic helpers due to a lack of exposure and training might have contributed to increased obesity/overweight risks in children under their care [33]. In all, higher adiposity among PCN children could be attributed to an overindulgence of grandparents and/or a lack of awareness of health recommendations among grandparents and domestic helpers.

Chen et al. reported higher sedentary behaviour on non-school days as compared to school days among preschool children in Singapore, with similar results using parent-reported data (4.0 vs. 2.8 h/day) and objectively measured using wrist-worn accelerometers (8.2 vs. 7.7 h/day) [50]. The authors attributed greater engagement in sedentary behaviour on non-school days to more time spent on screen devices [50]. Findings from this present study revealed likewise, with PCP and PCN children spending more time in sedentary behaviour compared to FC children, possibly explained by increased screen time. A Canadian study found that childcare centre attendance was negatively associated with screen time among toddlers (aged 19.0 ± 1.9 months) when compared to parental care, with findings more prevalent in ethnic minority groups [51].

Singapore’s national physical activity guidelines advised that children under the age of 7 should engage in at least 180 min of physical activity spread throughout the day [52]. In this study, physical activity levels of preschool children did not meet the guidelines and were not significantly different across childcare types. Similarly, Chen et al. reported that MVPA was consistently low among preschool children across school and non-school days [50]. In the childcare centre environment, cultural influences on academic performance might have resulted in the over-emphasis of intellectual development, thereby limiting participation in physical activities [50]. The home environment, on the other hand, supported sedentary behaviour instead of a physically active environment, with a median of seven screen devices per household [50]. Hence, regardless of the environment there is a general lack of MVPA among preschool children in Singapore. This supports our observation of low MVPA levels among FC, PCP and PCN children. Another study conducted in Belgium also reported that MVPA was consistently low among preschool children and were not significantly different on weekends and weekdays [53]. These findings reveal a need to prioritise and increase physical activity levels in both the childcare and home environments.

Many studies have reported the protective effects of parental care against childhood overweight/obesity [18,19,33,34], except studies conducted among lower-income groups where parental care was positively associated with childhood overweight/obesity instead [25,29]. The protective effects of parental care were not evident in our study, with FC children having lower adiposity instead. Although our findings revealed that children in PCP consumed higher amounts of whole grains, they tended to snack more throughout the day and consumed higher amounts of deep-fried food. Besides dietary factors, sociodemographic factors might have contributed to this finding, as mothers of PCP children were mainly non-professionals with a lower monthly income. Hence, they might have a lower health literacy and are less aware of certain dietary and physical activity guidelines.

### Strengths and Limitations

To our knowledge, this is the first study in Singapore to associate childcare arrangements in preschool children with adiposity measures, with further evidence from dietary, physical activity and sedentary behaviour measures. While most studies in other countries examine only one form of childcare (either parental, informal or centre-based), mixed forms of childcare (with parents and non-parents as caregivers) were examined in this study.

The limitations of this study are firstly, dietary, physical activity and sedentary behaviour measures were obtained from parental reports, and not from the respective caregivers themselves, hence parents might have misreported the data collected. Secondly, dietary, physical activity and sedentary behaviours were self-reported and not measured objectively. Thirdly, as this is a cross-sectional study, we were not able to test for causality and direction of relationship. Lastly, residual confounding might be present since other potential mediators that might affect adiposity measures, such as a child’s sleep duration and quality, child’s psychological characteristics, parenting styles, feeding practices and family lifestyle [7,54,55,56,57], were not examined in this study.

## 5. Conclusions

In conclusion, higher adiposity and greater likelihood of being overweight/obese was observed among children in partial centre-based childcare, especially in those who had non-parents as primary caregivers, compared to children in full-time centre-based childcare. The former group also had more screen time and greater consumption of unhealthy food.

While current policies focus mainly on parental and centre-based childcare interventions, our findings suggest that greater engagement of non-parental caregivers such as grandparents and domestic helpers are equally important. Additionally, more childcare centres should be encouraged to participate in government-initiated healthy eating programmes. Furthermore, greater engagement in physical activity should be encouraged in both home and childcare settings. Lastly, longitudinal studies should be carried out to further elucidate the strength of association between correlates of obesity and adiposity outcomes.

## Figures and Tables

**Table 1:** Characteristics of children and mothers (*n* = 540).

	Childcare Type			*p*-Value
	FC (*n* = 272)	PCP (*n* = 192)	PCN (*n* = 76)	
Child characteristics				
Sex				0.629
Male	148 (54.4)	96 (50.0)	41 (53.9)	
Female	124 (45.6)	96 (50.0)	35 (46.1)	
Birth order				0.002
First child	128 (47.1) ^a^	64 (33.3) ^b^	41 (53.9) ^a^	
Second child and above	144 (52.9) ^a^	128 (66.7) ^b^	35 (46.1) ^a^	
Childcare commencement (months)				<0.001
Between 2 and 24 months	145 (53.3) ^a^	23 (12.0) ^b^	15 (19.7) ^b^	
Between 25 and 60 months	127 (46.7) ^a^	169 (88.0) ^b^	61 (80.3) ^b^	
Birth weight (kg)	3.2 ± 0.4	3.1 ± 0.4	3.1 ± 0.5	0.155
Sum of skinfold thicknesses at birth (mm)	10.5 ± 2.1	10.3 ± 2.2	10.0 ± 2.3	0.136
Maternal characteristics				
Age (years)	31.0 ±5.4 ^a,b^	31.8 ± 5.0 ^a^	30.1 ± 5.0 ^b^	0.040
Ethnicity				<0.001
Chinese	195 (71.7) ^a^	78 (40.6) ^b^	43 (56.6) ^c^	
Malay	49 (18.0) ^a^	63 (32.8) ^b^	17 (22.4) ^a,b^	
Indian	28 (10.3) ^a^	51 (26.6) ^b^	16 (21.1) ^b^	
Highest education level				0.015
Secondary or lower	69 (25.4) ^a^	72 (37.5) ^b^	14 (18.4) ^a^	
Post-Secondary	101 (37.1)	59 (30.7)	32 (42.1)	
University or above	102 (37.5)	61 (31.8)	30 (39.5)	
Employment				<0.001
Employed	209 (76.8) ^a^	95 (49.5) ^b^	70 (92.1) ^c^	
Unemployed	63 (23.2) ^a^	97 (50.5) ^b^	6 (7.9) ^c^	
Household income				0.037
<$2000	34 (12.5) ^a^	40 (20.8) ^b^	7 (9.2) ^a^	
$2000–$5999	147 (54.0)	103 (53.6)	44 (57.9)	
>$6000	91 (33.5)	49 (25.5)	25 (32.9)	
Total physical activity at 6 years postpartum (min/week)				0.019
0	108 (39.7) ^a^	55 (28.6) ^b^	25 (32.9) ^a,b^	
>0–149	107 (39.3)	72 (37.5)	32 (42.1)	
≥150	57 (21.0) ^a^	65 (33.9) ^b^	19 (25.0) ^a,b^	
TV viewing at 6 years postpartum (min/week)				0.094
<60	143 (52.6)	81 (42.2)	43 (56.6)	
60–120	95 (34.9)	76 (39.6)	24 (31.6)	
>120	34 (12.5)	35 (18.2)	9 (11.8)	
Weight status at 6 years postpartum (BMI)				0.004
Underweight/normal (<23 kg/m^2^)	135 (49.6) ^a^	67 (34.9) ^b^	38 (50.0) ^a^	
Overweight/obese (≥23 kg/m^2^)	137 (50.4) ^a^	125 (65.1) ^b^	38 (50.0) ^a^	

FC: Full-time centre-based childcare; PCP: Partial centre-based childcare—Parent; PCN: Partial centre-based childcare—Non-parent. Values are expressed as *n* (%) or mean ± SD. ^a,b^ Values in the same row not sharing the same superscript are significantly different from one another using a two-sided test of equality for column proportions. Results were analysed using Pearson’s chi-square test and one-way analysis of variance (ANOVA) with Bonferroni post-hoc test. Imputed variables: Total skinfold at birth (*n* = 21); Maternal education (*n* = 3); Maternal employment (*n* = 12); Household income (*n* = 34); Maternal physical activity 6 years postpartum (*n* = 23); Maternal TV viewing 6 years postpartum (*n* = 24); Maternal body mass index (BMI) 6 years postpartum (*n* = 47).

**Table 2:** Comparison of dietary measures across childcare arrangements at age 5 (*n* = 540).

	Childcare Type			*p*-Value
	FC (*n* = 272)	PCP (*n* = 192)	PCN (*n* = 76)	
Food group, median (IQR)				
Fruits (g/day)	87.4 (103.0)	85.4 (123.0)	77.5 (85.9)	0.079
Vegetables (g/day)	26.7 (41.7)	26.3 (49.6)	24.8 (41.1)	0.622
Whole grains (g/day)	0.0 (23.7) ^a^	11.2 (45.0) ^b^	0.6 (36.9) ^a,b^	0.001
Deep-fried food (g/day)	17.5 (23.4) ^a^	25.7 (32.0) ^b^	22.1 (33.0) ^b^	0.003
Fast food (g/day)	12.9 (18.1) ^a^	15.7 (21.9) ^a,b^	17.9 (30.0) ^b^	0.035
Sweet snacks (g/day)	27.2 (33.1)	29.7 (37.8)	32.7 (39.1)	0.145
Sugar-sweetened beverages (mL/day)	113.5 (142.5)	110.2 (156.5)	115.1(124.3)	0.833
Eating out frequency, *n* (%)				0.314
At least once per day	15 (5.5)	9 (4.7)	3 (3.9)	
At least once per week	180 (66.2)	111 (57.8)	50 (65.8)	
At least once per month or rarely or never	77 (28.3)	72 (37.5)	23 (30.3)	
Snacking frequency, *n* (%)				0.030
Snacks all day but has meals	35 (13.0) ^a^	46 (24.0) ^b^	17 (22.7) ^b^	
Snacks only during tea time	170 (63.2)	111 (57.8)	44 (58.7)	
Does not snack much	64 (23.8)	35 (18.2)	14 (18.7)	

FC: Full-time centre-based childcare; PCP: Partial centre-based childcare—Parent; PCN: Partial centre-based childcare—Non-parent. Values for food groups are expressed as median (IQR) and values for eating out and snacking frequencies are expressed as *n* (%). ^a,b^ Values in the same row not sharing the same superscript are significantly different from one another using a two-sided test of equality for column proportions. Results were analysed using Kruskal–Wallis test and Pearson’s chi-square test. Values were removed for the “snacks all day and has no meals” option due to a small number (*n* = 4).

**Table 3:** Comparison of movement behaviours across childcare arrangements at age 5.5 (*n* = 540).

	Type of Childcare			*p*-Value
	FC (*n* = 272)	PCP (*n* = 192)	PCN (*n* = 76)	
Physical activity measures				
Total physical activity (min/day)	121.8 (140.7)	107.1 (146.3)	125.4 (141.4)	0.284
Total MVPA (min/day)	59.9 (91.4)	50.7 (91.1)	50.4 (92.7)	0.725
Sedentary behaviour measures				
Total sedentary behaviour (min/day)	187.9 (136.1) ^a^	216.8 (184.6) ^b^	260.4 (176.8) ^b^	0.005
Total screen time (min/day)	68.6 (83.6) ^a^	77.1 (119.8) ^a^	115.7 (117.5) ^b^	<0.001

FC: Full-time centre-based childcare; PCP: Partial centre-based childcare—Parent; PCN: Partial centre-based childcare—Non-parent; MVPA: Moderate-to-Vigorous Physical Activity Values are expressed as median (IQR). ^a,b^ Values in the same row not sharing the same superscript are significantly different from one another using a two-sided test of equality for column proportions. Results were analysed using Kruskal–Wallis test.

**Table 4:** Associations of childcare arrangements at age 5 and adiposity measures at age 6 (*n* = 540).

	Unadjusted Model			Adjusted Model		
	*β*	95% CI	*p*-Value	*β*	95% CI	*p*-Value
BMI *z*-score						
FC	Reference			Reference		
PCP	0.23	−0.001, 0.46	0.051	0.12	−0.15, 0.38	0.387
PCN	0.38	0.07, 0.70	0.016	0.34	0.01, 0.66	0.042
Sum of skinfold thicknesses						
FC	Reference			Reference		
PCP	2.46	0.19, 4.73	0.033	0.88	−1.75, 3.51	0.511
PCN	4.46	1.34, 7.58	0.005	3.75	0.53, 6.97	0.022

FC: Full-time centre-based childcare; PCP: Partial centre-based childcare—Parent; PCN: Partial centre-based childcare—Non-parent. Results were analysed using the multivariable general linear model. BMI *z*-scores and sum of skinfold thicknesses were adjusted for age of childcare commencement, birth order, ethnicity, maternal age, maternal education, maternal BMI 6 years postpartum and maternal physical activity level 6 years postpartum.

**Table 5:** Associations of childcare arrangements at age 5 and adiposity status at age 6 (*n* = 540).

	Overweight/Obese		
	Adjusted Odds Ratio	95% CI	*p*-Value
FC	Reference		
PCP	1.54	0.83, 2.87	0.169
PCN	3.55	1.78, 7.05	<0.001

FC: Full-time centre-based childcare; PCP: Partial centre-based childcare—Parent; PCN: Partial centre-based childcare—Non-parent. Results were analysed using the binary logistic regression model. The model was adjusted for age of childcare commencement, birth order, ethnicity, maternal age, maternal education, maternal BMI 6 years postpartum and maternal physical activity level 6 years postpartum. Underweight/normal weight was the reference category.

## Data Availability

The datasets generated and/or analysed during the current study are not publicly available due to an ethical restriction (patient confidentiality) which was imposed by the Centralised Institutional Review Board of SingHealth. Interested researchers may request the data by contacting the data team leader of GUSTO at Raja_Sunil_Kumar@sics.a-star.edu.sg.

## Supplementary Materials

The following are available online at https://www.mdpi.com/article/10.3390/ijerph182212178/s1, Table S1: Breakdown of food groups; Table S2: Breakdown of primary caregiver identity among childcare centre-going participants (*n* = 540); Table S3. Comparison of demographic characteristics between included and excluded eligible participants (*n* = 1237).
