# Supplementary material for: Associations of Childcare Arrangements with Adiposity Measures in a Multi-Ethnic Asian Cohort: The GUSTO Study
Source: Int J Environ Res Public Health. 2021 Nov 19;18(22):12178. doi: 10.3390/ijerph182212178 (PMC8622483; doi:10.3390/ijerph182212178)
Supplement: Supplementary file 1 [file ijerph-18-12178-s001.zip › ijerph-1453330-supplementary.pdf]

Table S1. Breakdown of food groups

Food group	List of food items
Fruits	Dried fruits, guava, dragonfruit, melon, tropical fruit, stone fruit, grapes and berries, orange, papaya, banana, apple and pears.
Vegetables	Mushroom, gourd, lentils, stalk vegetables, broccoli, pale and dark leafy vegetables, tomato, red and green peppers, corn, peas and beans, carrot, pumpkin.
Whole grains	Unpolished rice, unpolished rice porridge, oats, wholemeal bread/
Deep fried food	Dimsum fried, eggs fried, oily fish and non-oily fish deep fried, pork and chicken deep fried, pan fried bread.
Fast food	Fried potatoes, chicken/fish batter, pizza, burger.
Sugar-sweetened beverages (SSB)	Milo, Horlicks, regular and reduced sugar cultured drinks, sweetened drinks, low calorie drinks, soya milk, traditional drinks.
Sweet snacks	Ice cream, jellies, pudding, sweets, chocolate, fried doughy snacks, biscuits and cookies, puff pastries, butter and cream cakes, sponge and steamed cakes.

Table S2. Breakdown of primary caregiver identity among childcare centre-going participants (n = 540)

Childcare type	Primary caregiver identity	N (%)	Total N (%)
FC	Childcare centre establishment/ childcare centre teachers	272 (100.0)	272 (50.4)
PCP	Mother	185 (96.4)	192 (35.5)
	Father	5 (2.6)	
	Both parents	2 (1.0)	
PCN	Grandparent	39 (51.3)	76 (14.1)
	Domestic helper	33 (43.4)	
	Others (Aunty)	2 (2.6)	
	Both non-parents (grandparent and helper, n=1; childcare and grandparent, n=1)	2 (2.6)	

FC: Full-time centre-based Childcare; PCP: Partial centre-based Childcare – Parent; PCN: Partial centre-based Childcare – Non-parent.

Table S3. Comparison of demographic characteristics between included and excluded eligible participants (n=1237)

	Included (n = 540)	Excluded (n = 697)	P-value
Child characteristics			
Sex			0.893
Male	285 (52.8)	368 (52.8)	
Female	255 (47.2)	329 (47.2)	
Birth order			0.090
First child	233 (43.1)	334 (47.9)	
Second child and above	307 (56.9)	363 (52.1)	
Age of childcare commencement (months)			0.036
Between 2 and 24 months	183 (33.9)	198 (28.4)	
Between 25 and 60 months	357 (66.1)	499 (71.6)	
Birth weight (kg)	3.1 ± 0.4	3.0 ± 0.5	0.003
Sum of skinfold thicknesses at birth (mm)	10.4 ± 2.2	10.3 ± 2.4	0.488
Maternal characteristics			
Age at recruitment (years)	31.1 ± 5.2	30.0 ± 5.0	<0.001
Ethnicity			0.219
Chinese	316 (58.5)	375 (53.8)	
Malay	129 (23.9)	193 (27.7)	
Indian	95 (17.6)	129 (18.5)	
Highest education level			0.144
Secondary or lower	155 (28.7)	233 (33.4)	
Post-Secondary	192 (35.6)	244 (35.0)	
University or above	193 (35.7)	220 (31.6)	
Employment			0.441
Employed	374 (69.3)	497 (71.3)	
Unemployed	166 (30.7)	200 (28.7)	
Household income			0.482
<$2000	81 (15.0)	113 (16.2)	
$2000-$5999	295 (54.6)	392 (56.2)	
>$6000	164 (30.4)	192 (27.6)	
Total physical activity at 6 years postpartum (min/day)			0.369
0	187 (34.6)	258 (37.0)	
>0-149	211 (39.1)	273 (39.2)	
≥ 150	142 (26.3)	166 (23.8)	
TV viewing time at 6 years postpartum (min/day)			0.252
<60	267 (49.4)	333 (47.8)	
60-120	195 (36.1)	243 (34.9)	
>120	78 (14.5)	121 (17.3)	
Weight status at 6 years postpartum (BMI)			0.350
Underweight/normal (< 23 kg/m^2)	240 (44.4)	295 (42.3)	
Overweight/obese (≥23 kg/m^2)	300 (55.6)	402 (57.7)	

Values are expressed as n (%) or mean ± SD. Results were analysed using Pearson's chi-square test and independent samples t-test.

Table S4. Comparison of demographic characteristics between participant with and without childcare arrangement data (n=1237)

	With Data (n = 876)	Without Data (n = 361)	P-value
Child characteristics			
Sex			0.746
Male	461 (52.6)	191 (52.9)	
Female	415 (47.4)	170 (47.1)	
Birth order			0.398
First child	394 (45.0)	172 (47.6)	
Second child and above	482 (55.0)	189 (52.4)	
Age of childcare commencement (months)			<0.001
Between 2 and 24 months	298 (34.0)	85 (23.5)	
Between 25 and 60 months	578 (66.0)	276 (76.5)	
Birth weight (kg)	3.1 ± 0.4	3.0 ± 0.5	0.001
Sum of skinfold thicknesses at birth (mm)	10.4 ± 2.2	10.1 ± 2.5	0.027
Maternal characteristics			
Age at recruitment (years)	30.9 ± 5.1	29.6 ± 5.1	<0.001
Ethnicity			0.283
Chinese	499 (56.9)	192 (53.2)	
Malay	217 (24.8)	105 (29.1)	
Indian	160 (18.3)	64 (17.7)	
Highest education level			0.013
Secondary or lower	259 (29.6)	128 (35.5)	
Post-Secondary	303 (34.6)	134 (37.1)	
University or above	314 (35.8)	99 (27.4)	
Employment			0.631
Employed	620 (70.8)	250 (69.3)	
Unemployed	256 (29.2)	111 (30.7)	
Household income			0.228
<$2000	133 (15.2)	62 (17.2)	
$2000-$5999	479 (54.7)	207 (57.3)	
>$6000	264 (30.1)	92 (25.5)	
Total physical activity at 6 years postpartum (min/day)			0.178
0	311 (35.5)	135 (37.4)	
>0-149	348 (39.7)	135 (37.4)	
≥ 150	217 (24.8)	91 (25.2)	
TV viewing time at 6 years postpartum (min/day)			0.111
<60	429 (49.0)	171 (47.4)	
60-120	311 (35.5)	127 (35.2)	
>120	136 (15.5)	63 (17.4)	
Weight status at 6 years postpartum (BMI)			0.318
Underweight/normal (< 23 kg/m^2)	381 (43.5)	154 (42.7)	
Overweight/obese (≥23 kg/m^2)	495 (56.5)	207 (57.3)	

Values are expressed as n (%) or mean ± SD. Results were analysed using Pearson's chi-square test and independent samples t-test.
